# Vitamins in Human and Donkey Milk: Functional and Nutritional Role

**DOI:** 10.3390/nu13051509

**Published:** 2021-04-29

**Authors:** Silvia Vincenzetti, Giuseppe Santini, Valeria Polzonetti, Stefania Pucciarelli, Yulia Klimanova, Paolo Polidori

**Affiliations:** 1 School of Biosciences and Veterinary Medicine, University of Camerino, 62032 Camerino, Italy; silvia.vincenzetti@unicam.it (S.V.); giuseppe.santini@unicam.it (G.S.); valeria.polzonetti@unicam.it (V.P.); stefania.pucciarelli@unicam.it (S.P.); yulia.klimanova@unicam.it (Y.K.); 2 School of Pharmacy, University of Camerino, 62032 Camerino, Italy

**Keywords:** vitamins, donkey milk, human milk, vitamins deficiency, fortified foods

## Abstract

Background: Whole milk is a good source of all the nutrients, and it also contains a sufficient number of vitamins to permit regular the growth of the neonate. Dairy cow milk can create allergy in infants less than 12 months old because of the high caseins and β-lactoglobulin content. In these circumstances, donkey milk can represent a good replacement for dairy cows’ milk in children affected by Cow Milk Protein Allergy (CMPA) because of its close chemical composition with human milk, mainly due to its low protein and low mineral content. Milk vitamin content is highly variable among mammalian species and it is strictly correlated with the vitamin status and the diet administered to the mother. Fat-soluble vitamins content in donkey milk is, on average, lower compared to ruminants’ milk, while vitamin C content determined in donkey milk is higher compared to dairy cows’ milk, showing a great similarity with human milk. In donkey milk, the content of vitamins of the B-complex such as thiamine, riboflavin, niacin, pyridoxine, and folic acid is higher compared to human milk. The use of donkey milk as a new functional food must be further evaluated in interdisciplinary clinical trials in which pediatricians, dietitians, and food scientists must be involved to deepen the knowledge about the positive health impact of donkey milk in different sensitive people, especially children and the elderly.

## 1. Introduction

The production of animal source foods through the breeding of domesticated species represents a milestone in the development of human civilizations. Milk is the most important food for newborns in all mammalian species, supplying energy, proteins, fat, carbohydrates, and micronutrients, such as vitamins and minerals. Milk consumption started with animal domestication about 10,000 years ago, the advantages of which were mainly related to the positive effects on growth and bone health [1]. The term “milk” is normally associated with bovine milk because it represents 83% of global milk production in 2010 [2].

However, milk produced from other animal species is also consumed. Equid milk, obtained both from mare (*Equus caballus*) and donkey (*Equus asinus*), shows remarkably interesting therapeutic properties, especially in the treatment of Cow Milk Protein Allergy (CMPA) in infant nutrition [3]. Clinically, CMPA is an abnormal immunological reaction to cow milk proteins, normally caused by the interaction between one or more milk proteins and one or more immune mechanisms, provoking immediate IgE-mediated reactions. The clinical diagnosis CMPA can be achieved by skin or blood tests, and the incidence of CMPA in children ranges from 0.3 to 7.5% [3]. Non-IgE-mediated dairy reactions occur at variables time after milk exposure. These reactions are significantly more common compared to IgE-mediated immune reactions and are caused by a T-cell-mediated reaction to cow milk proteins, sometimes through a combination of both IgE- and non-IgE-mediated reactions. The allergenicity of cow milk in children’s nutrition is basically due to the ratio between caseins and whey proteins. Variations in the ratio between caseins and whey proteins in donkey milk are considered the main factor responsible for reducing the allergenicity of bovine milk [3].

A significant part of the population in Russia, Kazakhstan, Kyrgyzstan, Tajikistan, Uzbekistan, and Mongolia drink mare milk [4]. Milk produced by these two monogastric species shows similar protein, fat, lactose, ash, and water content (Table 1), with a chemical composition very close to that in human milk, considering the high lactose, low protein, and ash content [5].

The low caseins content in equine milks, around 40%–45% of total protein, is very close to the values determined in human milk (Table 2). In these two mammalian species, more than 50% percent of milk protein is represented by whey protein (Table 2), and for this reason, cheesemaking using equid milk is challenging [7,8,9]. Among whey proteins, a total amount of 1.0 mg/mL of lysozyme has been detected in donkey milk, while in bovine milk, a very reduced amount (trace) has been determined [10]. Although β-lactoglobulin is present in the whey proteins of donkey milk, the sequence homology determined among β-lactoglobulin isolated from both donkey and cow milk is only 60 percent [11]. In 1990, the high similarity of milk proteins detected in both equine and human milk created the opportunity to test donkey milk in the treatment of children affected by severe IgE-mediated CMPA [12].

The growth rate in human infants is slower compared to other animals, and the chemical composition of human milk reflects this [6]. The similar chemical composition determined among equine and human milk (Table 1 and Table 2) suggests that donkey milk can be used as a dietary alternative for children with IgE- and non-IgE-mediated CMPA when breast milk is not available. Bovine milk is considered the main replacement for human milk in infant nutrition, but it is remarkably different from human milk considering both macronutrients and micronutrients, with different contents of both vitamins and minerals [8,12]. Vitamin content in donkey milk has not been deeply investigated compared to its protein fraction or fat profile. The aim of the present review was to compare the vitamin content in human and donkey milk, to evaluate the nutritional properties of donkey milk in order to better understand the suitability of this food in infant’s nutrition and in other consumers category such as elderly people.

## 2. Nutrition and Health: Role of Milk and Dairy Products

Consumption of animal source foods can provide crucial nutritional benefits to a large amount of the population in developing countries, where most of the consumers cannot afford good-quality diets due to their low income. However, the rapid growth in animal-sourced food production and consumption may represent a risk for human and animal health, and for the environment as well. On the other hand, it can also offer opportunities for local farmers and small dairy industries. Milk provides several nutrients and can help consumers cover the daily requirements for minerals and vitamins [13].

Milk and dairy products are considered very important foods for healthy human nutrition, especially in childhood [14]. Milk is a complete food containing fat, proteins, carbohydrates, minerals, and vitamins. Some studies have evidenced the role of dairy foods as important macro- and micronutrient sources, considering their presence in a healthy diet to be positive [14]. Milk consumption is frequently associated with a reduction of the risk of osteoporosis, colorectal cancer, and of type 2 diabetes, but high amounts of dairy foods in the human diet have been reported to be responsible for increasing cardiovascular disease and prostate cancer [15]. Milk is also considered important in reducing moderate malnutrition in children, together with other dairy products, especially fermented milk (yoghurt), which is characterized by a similar chemical composition compared to fresh milk, apart from the reduced lactose content [16].

Food hypersensitivity is the most common cause of reduced absorption and slow growth rates in lactating children after weaning. Between 2%–7.5% of infant population is affected by CMPA, with an increased incidence compared to 1940, when 1 positive case was found for every 7500 subjects tested. Today, 1 positive case is detected for 200 subjects tested [17]. Remarkable progress has been registered in parenteral nutrition, significantly improving accuracy in prognosis, even if the need to restart feeding process as soon as possible is considered the most important target to stimulate the correct functions of the damaged intestine [18]. Breastfeeding is the best strategy to achieve this target, but if human milk is not immediately available, it is necessary to look for other foods. Beverages containing soy formulas or hydrolyzed proteins can be responsible of severe hypersensitivity reactions [19]. Since 1992, several clinical trials have demonstrated that donkey milk can be safely used in the treatment of multiple food allergies, including CMPA [20]. The nutraceutical properties of donkey milk have been investigated in the last decades, finding important bioactive molecules with antimicrobial and antiallergenic functions [5]. 

Both caseins and whey proteins contribute to human health, performing a biological activity after enzymatic digestion. The molecules released after enzymatic digestion are named bioactive peptides because of their ability to modulate physiological functions [10,11]. In donkey milk, several bioactive peptides showing immunological-like properties with the ability to stimulate the functional recovery of the child’s intestine have been determined, such as lysozyme, lactoperoxidase, and lactoferrin. These enzymes are active against protozoa, bacteria, and viruses, and are therefore also able to prevent infections in children’s intestine [10,11].

It is outside the aim of this review to describe, in detail, bioactive peptides, specific studies on the bioactive molecules detected in donkey milk, and their positive effects on human health [3].

## 3. Water-Soluble Vitamins

The authors of [21] investigated the vitamin content in milk obtained from minor dairy species, showing a substantial contribution provided by both mare and camel milk to the recommended human daily requirements for vitamin B-complex and vitamin C [21]. In particular, vitamin B_12_ (Cobalamin) is naturally provided only in foods of animal origin. In fact, it is commonly deficient in people who have a diet poor in animal-sourced foods.

In Table 3, the recommended nutrients intake for children younger than 3 years is listed for both water-soluble and fat-soluble vitamins [21].

The vitamin C level determined in donkey milk (57 mg/L) is very close (Table 4) to the content reported in human milk (60 mg/L). The important roles performed by the eight B-complex water-soluble vitamins involve several cellular functions, in which they work as coenzymes in different catabolic and anabolic enzymatic reactions. Vitamin B_6_ content in donkey milk has been recently determined for the first time [22], together with other B-complex vitamins, such as thiamine, riboflavin, and nicotinic acid, characterized by a lipid-lowering effect, and folic acid, which is particularly important for children’s growth [23].

As shown in Table 4, the thiamine concentration in donkey milk (0.66 µM) has a very high value compared to that determined in human milk (0.12 µM) [24]. Riboflavin concentration in donkey milk (0.17 µM) is higher with respect to the value reported in human milk (0.08 µM), but it is very low compared to the values obtained both in cow and goat milk [24,25]. Riboflavin is the precursor of two cofactors that are biologically involved in the oxidation-reduction reactions, specifically flavin adenine mononucleotide (FMN) and flavin adenine dinucleotide (FAD). The high contents of both thiamine and riboflavin determined in donkey milk have been described as one of the causes of the health effects associated with this food when administered in human nutrition [26,27].

As shown in Table 4, the nicotinic acid (Vitamin B_3_) content in donkey milk shows a value higher (18.75 µM) compared to human (4.64 µM) and cow milk but a value similar to that reported in goat milk [28]. Nicotinic acid, also named niacin, is a vitamin able to perform lipid-lowering effects, lowering both triglycerides and serum cholesterol [22].

Vitamin B_6_ (pyridoxine) content (5.38 µM) in donkey milk (Table 4) is very high compared to human milk (0.48 µM) but close to the value determined in cow milk [29]. Vitamin B_6_ is involved in several biochemical pathways, such as amino acid metabolism, lipid metabolism, and gluconeogenesis. Lack of vitamin B_6_ is common, especially among the elderly and in fertile-aged women, leading to reduced immune responses [30].

Milk represents an important source of folic acid (Vitamin B_9_) for children younger than 1 year of age. Folic acid content in donkey milk (Table 4) is higher (0.83 µM) compared to human milk (0.37 µM). Folic acid is involved in nucleic acid synthesis. In pregnant women, folic acid plays a key role in the growth and development of the fetus [23]. Lack of vitamin B_9_ can be responsible for several diseases in children (neural tube defects), adults (megaloblastic anemia, cardiovascular disease, cancer), and the elderly (Alzheimer’s disease) [23].

Vitamin B_12_ (cyanocobalamin) was not detected in donkey milk, confirming the findings obtained when analyzing donkey milk collected by Indian small grey donkeys [31]. The lack of vitamin B_12_ in equid milk compared to ruminant milk could be explained by the different digestive systems among these two mammalian species, which are both herbivores. Vitamin B_12_ is synthesized by the microorganisms of the digestive tract. Donkey is a hind gut fermenter, while in ruminants, most of the fermentations are developed in the rumen.

Vitamin C is an important antioxidant and free radical scavenger [32]. Fresh donkey milk contains 57 mg/L of vitamin C (Table 4). This value is very close to that determined for human milk (60 mg/L) and is significantly higher compared with the vitamin C content in cow milk [33]. In other animals living in arid and semiarid areas, such as camels, the amount of vitamin C determined in their milk is very close to that found in donkey milk, which has been linked to the poor availability of green vegetables and fruits for human and animal nutrition [34]. Vitamin C plays a role in intestinal iron absorption and is essential for collagen formation. In high-risk children, vitamin C demonstrates a protective effect against atopic dermatitis as well [33]. When administered to children younger than 1 year of age, donkey milk represents a good source of vitamin C. In fact, 500 mL of donkey milk provides the recommended daily intake (30 mg) for 0–12-month-old children [35].

**Table 4:** Water-soluble vitamins content in human, cow, and donkey milk (µM).

Water-Soluble Vitamin	Donkey Milk	Cow Milk	Human Milk
Thiamine (vitamin B_1_)	0.66	0.59	0.12
Riboflavin (V1itamin B_2_)	0.17	2.12	0.08
Niacin (Vitamin B_3_)	18.75	2.43	4.64
Piridoxine (Vitamin B_6_)	5.38	5.50	0.48
Folic acid (Vitamin B_9_)	0.83	0.02	0.37
Cyanocobalamin (Vitamin B_12_)	n.d.	3.3 × 10^−3^	n.d.
Vitamin C [36]	57 mg/L	27 mg/L	60 mg/L

n.d.: not detected.

## 4. Fat-Soluble Vitamins

Animal’s diet has a great effect on the milk content of fat-soluble vitamins (A, D, E, and K). Significant differences can be detected in fat-soluble vitamin content in different kinds of milk according to the specific mammalian species. Within a specific species, the concentration of fat-soluble vitamin content in milk is strictly affected by animal breed. In dairy cows, breeds producing milk characterized by a high fat content, such as Jersey and Guernsey, show a higher content of fat-soluble vitamins compared to milk produced by other breeds, such as Friesian and Brown. There is a direct correlation between the fat-soluble vitamins determined in milk and the total milk fat content, with specific implications related to milk’s nutritional and sensorial properties [37]:

- In Western countries, milk provides an important percentage of the RDA (recommended daily allowance) for the fat-soluble vitamins. For this reason, in many countries, both milk and butter are frequently fortified with vitamins A and D.
- Dairy foods with high fat content are characterized by a yellow-orange color due to the carotenoids and vitamin A content, which mainly affected by animal nutrition.
- Milk from goats, sheep, buffalo, and donkeys shows low levels of carotenoids. For for this reason, milk from these animals is whiter compared to bovine milk.

Vitamin A is a molecule formed by active compounds, retinoids, and carotenoids [38]. Carotenoids are normally detected in vegetables. uring the digestive processes, carotenoids are converted into retinol. The conversion rate is different according to the specific animal digestive physiology. Then retinol from the liver is transferred to the mammary gland, where it is esterified and finally secreted in the milk fat globules [39].

Vitamin A in milk is particularly important in children’s nutrition, affecting newborn growth, immunity development, and eye health and playing a role in maintaining epithelial integrity [40]. Vitamin A content in milk is strictly correlated with milk’s total fat content, which is influenced by factors such as animal diet and season [41].

Vitamin A content (Table 5) is slightly higher in human milk (60 µg/100 mL) compared to donkey milk (58 µg/100 mL). Normally, in Western countries, there are no vitamin A deficiencies [35]. Human milk can be considered as a good vitamin A source, but in several countries, it is common practice to fortify dairy products with this vitamin to improve nutritional status and to provide a greater amount of vitamin A, particularly in children’s nutrition [42].

Vitamin D is a group of compounds deeply involved in calcium and bone metabolism, therefore performing an antirachitic activity in growing mammals and acting as a hormone [45]. Human milk contains a very low amount (0.06 µg/100 mL) of vitamin D (Table 4). In donkey milk, the total vitamin D level (2.23 µg/100 mL) is higher compared to the values determined in milk obtained in many other mammalian species, including human milk [46]. Recently, several clinical trials have investigated the anticarcinogenic, anti-celiac, and immunomodulatory properties of vitamin D, together with the well-known physiological role performed by this micronutrient in bone mass formation and osteoporosis prevention [47,48].

Vitamin E is a group of eight biologically active forms, represented by four tocopherols and four tocotrienols with antioxidant activity. In donkey milk, both α-tocopherol and γ-tocopherol have been detected [38]. Human milk is characterized by a high content of vitamin E (237 µg/100 mL) compared to donkey (5.2 µg/100 mL) and cow milk (Table 4). Vitamin E is one of the major natural antioxidants, demonstrating a protecting effect on cells membranes against oxidation or peroxidation processes [49].

According to published data, vitamin K has never been detected in donkey milk, while mare milk shows about three-times more vitamin K compared to cow milk. Moreover, human milk shows a very low level of vitamin K content [50].

Dietary intake of vitamins through milk and dairy foods has been deeply investigated [1]. Milk is an essential food in children’s nutrition from birth to weaning. Normally during this period, human milk is replaced with bovine milk, which provides a significant contribution to the recommended daily intake for several micronutrients, mainly vitamins A, D, B_2_, B_5_, and B_12_ [51]. Milk consumption has recently decreased all over the world, therefore reducing the dietary intake of vitamins through milk [52]. The human population registered a significant increase in recent decades because of the associated changes in dairy foods consumption, and the strategy of producing new fortified foods in order to provide the recommended vitamins daily intake in human nutrition has been developed. In the United States, the fortification of vitamin content in milk and dairy products is a common practice that requires specific approvals by the U.S. Food and Drug Administration [14].

Advances in knowledge with respect to the nutritional parameters of donkey milk, including the vitamin content, confirm the possibility of administering this nutraceutical food in diets for sensitive consumers. The high similarity among human and donkey milk chemical composition, also considering the associated levels of specific bioactive compounds determined in donkey milk, has drawn special attention to the use of this food in infant nutrition, especially in children affected by CMPA.

## 5. Clinical Trials Performed Using Donkey Milk in Children Affected by CMPA

The growth rate in children is slower than that in young animals of other mammalian species, except other primates [53], and human milk chemical composition reflects this physiological condition. When breast milk is not available, derived bovine milk formulas are normally administered to children as a replacement for human milk. However, cow milk is very different from human milk, considering both its macronutrients and micronutrients content. Human and donkey milk show different absorption rates of vitamins and minerals, with critical situations associated with infants [53]. When a child is affected by CMPA, the only successful therapy is based on the total removal of cow milk from the diet. Analyzing the strategy of administering milk from other mammalian species, equid milk is considered a valid replacement, from both mare and donkey, since their chemical composition is very similar to human milk (Table 1). However, the availability of mare milk is limited in specific regions in the world, particularly in the former Soviet Union Countries [54]. The phylogenetic relationship among donkeys and horses can be observed considering the similarity of chemical composition in each milk (Table 1).

Since 1990, the use of donkey milk in cases of CMPA has been evaluated in several clinical trials, attracting great scientific attention, especially among pediatric allergists [55,56]. The choice of a possible replacement for cow milk in cases of CMPA is based mainly on two factors, nutritional properties and allergenic characteristics, together with other important criteria, such as cost and taste. Patients showing an allergy to ruminant milk can normally tolerate donkey milk [57,58,59]. Although the mechanism for tolerance to donkey milk is still not completely understood, it is normally attributed to its different content of major allergenic molecules in milk, particularly the protein fractions affecting the caseins/whey proteins ratio [60]. In Table 6, the clinical studies in which donkey milk has been used in the treatment of CMPA are shown. Tolerability has been investigated in vivo and in vitro, performing clinical check-ups (IgE cross-reactivity versus donkey milk proteins) and auxological evaluations, namely weight and body length [59].

The first case of donkey milk allergy was reported in 2017 [64]. A 25-year-old woman, with a previous personal history of recurrent atopic dermatitis, allergic asthma, and hay fever, showed facial angioedema 5 min after her first intake of fresh donkey milk. She showed severe dyspnea, collapsing a few minutes after. A treatment based on adrenaline and corticosteroids was administered to the patient. Another case of donkey milk allergy was recently described in a 35-year-old woman who had never shown food allergies but had a cat allergy, seasonal allergic rhinitis, and asthma caused by grass and olive pollen. The woman developed a respiratory allergy after her intake of donkey milk, showing rhino conjunctivitis and asthma [65].

After several years in which different functional characteristics have been attributed to donkey milk, with clinical trials performed both in infant and elderly nutrition, recently, a group of scientists from North Africa used the term “pharma food,” for the first time for donkey milk. The term considers all of the positive results obtained in categories of consumers who demonstrate completely different nutritional requirements, such as children and/or adults and elderly people [66]. A limitation to the possible large-scale use donkey milk is represented by its low total production amount and, consequently, high cost on the market [67].

## 6. Conclusions

Donkey milk shows peculiar nutritional properties comparable to those determined in human milk. For this reason, its hypoallergenic properties, mainly due to the antimicrobial activity of several bioactive peptides, have been deeply investigated in the past years. Several factors can influence donkey milk’s chemical composition and its nutritional characteristics, including animal species, season, animal nutrition, lactation period, etc. Most of the biologic properties of donkey milk are not well known. Thus, many donkey milk compounds must be further investigated to permit a real development of the donkey milk industry. Interesting data regarding donkey milk vitamins content have been recently obtained, finding a considerable amount of both water-soluble vitamins, such as B-complex and vitamin C, as well as fat-soluble vitamins, particularly vitamin D. The vitamin content in donkey milk confirms the remarkable nutritional value of this food. Donkey milk is suitable for feeding children affected by CMPA and is also appropriate for elderly people.

## Figures and Tables

**Table 1:** Chemical composition in human, cow, and equid milk (g/100 g).

Mammal	Water	Dry Matter	Fat	Proteins	Lactose	Ash	Energy Value (kJ/kg)
Human	87.6	12.4	3.38	1.64	6.69	0.22	2855.6
Cow	87.6	12.4	3.46	3.43	4.71	0.78	2983.0
Donkey	90.4	9.61	1.21	1.74	6.23	0.43	1939.4
Mare	90.5	9.52	0.85	2.06	6.26	0.35	1877.8

Source: [6].

**Table 2:** Main proteins content in cow, donkey, and human milk (g/L).

Proteins	Cow	Donkey	Human
Total proteins	32.0	13–18	9–15
Total caseins	27.2	6.6	5.6
Total whey proteins	4.5	6.5	8.0
αs1-casein	10.0	n.d.	0.8
αs2-casein	3.7	n.d.	n.d.
β-casein	10	n.d.	4.0
k-casein	3.5	Trace	1.0
α-lactalbumin	1.2	1.80	1.9–2.6
β-lactoglobulin	3.3	3.7	n.d.
Lysozyme	Trace	1.0	0.04–0.2
Lactoferrin	0.1	0.08	1.7–2.0
Immunoglobulins	1.0	n.d.	1.1
Albumin	0.4	n.d.	0.4

n.d.: not detectable; source: [3].

**Table 3:** Recommended vitamins intake for children of different ages.

Fat-Soluble Vitamins	7–12 Months	1–3 Years
Vitamin A	400 µg/day	400 µg/day
Vitamin D	5 µg/day	5 µg/day
Vitamin E	2.7 mg/day	5 mg/day
Vitamin K	10 µg/day	15 µg/day
Water-Soluble Vitamins		
Vitamin B_1_	0.3 mg/day	0.5 mg/day
Vitamin B_2_	0.4 mg/day	0.5 mg/day
Vitamin B_3_	4 mg/day	6 mg/day
Vitamin B_5_	1.8 mg/day	2 mg/day
Vitamin B_6_	0.3 mg/day	0.5 mg/day
Vitamin B_8_	6 µg/day	8 µg/day
Vitamin B_9_	80 µg/day	150 µg/day
Vitamin B_12_	0.7 µg/day	0.9 µg/day
Vitamin C	30 mg/day	30 mg/day

**Table 5:** Fat-soluble vitamins content in human, cow, and donkey milk.

Fat-Soluble Vitamin	Human Milk [43]	Cow Milk [43]	Donkey Milk [44]
Vitamin A	60 µg/100 mL	41 µg/100 mL	58 µg/100 mL
Vitamin D	0.06 µg/100 mL	0.08 µg/100 mL	2.23 µg/100 mL
Vitamin E	237 µg/100 mL	113 µg/100 mL	5.2 µg/100 mL
Vitamin K	0.2 µg/100 mL	1.1 µg/100 mL	n.d.

n.d.: Not detected.

**Table 6:** Clinical studies on the tolerability of donkey milk in children with CMPA.

Children (*n*)	Age (Months Range)	Tolerability (%)	Reference
9	0–3	100	[20]
21	2–3	86	[58]
46	1–146	82.6	[59]
28	6–36	88	[19]
25	6–11	96	[61]
92	7.5–121.5	90.2	[60]
6	4.3	100	[12]
70	62	94.3	[62]
22	9–79	100	[63]
